# Associations of sleep quality and physical activity with anxiety and depressive symptoms among university students: a cross-sectional study

**DOI:** 10.3389/fpubh.2026.1799311

**Published:** 2026-03-19

**Authors:** Robinson Ramírez-Vélez, Juan Carlos Calderón-González, Luis Hebert Palma-Pulido, Andrés Felipe Mejía-Vásquez, Carolina Arias-Álzate, Juan Carlos Urriago-Fontal, Gustavo Adolfo Gutiérrez-Quintero, Julián David Ortiz-López, Diana Milena Bedoya-Salazar, Gonzalo Romero-Martínez, Mikel Izquierdo

**Affiliations:** 1 Facultad de Ciencias de la Educación, Unidad Central del Valle del Cauca (UCEVA), Túlua, Colombia; 2 Navarrabiomed, IdiSNA, Hospital Universitario de Navarra (HUN), Universidad Pública de Navarra (UPNA), Pamplona, Spain; 3 CIBER of Frailty and Healthy Aging (CIBERFES), Instituto de Salud Carlos III, Madrid, Spain

**Keywords:** Latin American, lifestyle behaviors, mental well-being, physical activity, sleep quality

## Abstract

Sleep disturbances and insufficient physical activity are common in university populations and have been associated with adverse mental health outcomes; however, evidence integrating these factors in Latin American student populations remains limited. This study examined the associations of sleep quality and physical activity with anxiety and depressive symptoms among university students and assessed whether physical activity moderates the association between sleep quality and mood symptoms. This cross-sectional analysis was conducted within the EpiHealth-UCEVA project and included 1,125 undergraduate students from a Colombian public university. Anxiety and depressive symptoms were assessed using the Hospital Anxiety and Depression Scale (HADS), sleep quality using the Pittsburgh Sleep Quality Index (PSQI), and physical activity using the International Physical Activity Questionnaire (IPAQ). Multivariable linear regression models, along with moderation analyses using PROCESS (Model 1), were conducted. In adjusted regression models, poorer sleep quality was associated with higher anxiety (*B* = 0.59) and depressive symptoms (*B* = 0.10). Moderate physical activity was associated with lower anxiety, whereas high physical activity was associated with lower depressive symptoms. In moderation analyses, the overall sleep × physical activity interaction was not statistically significant. These findings suggest complementary roles of sleep quality and physical activity in students’ mental health and highlight the potential value of integrated lifestyle-based strategies to support mental well-being in university settings.

## 1 Introduction

According to the Global Burden of Disease Study 2021, approximately 444 million incident cases of mental disorders were recorded worldwide in 2021, underscoring the substantial and persistent global burden of mental illness (1). Young adults appear to be particularly vulnerable, as many mental disorders first emerge during late adolescence and early adulthood. University students, who are navigating academic, social, and developmental transitions, consistently report elevated levels of psychological distress (2). In this context, a substantial proportion of university students experience depression, anxiety, and stress symptoms. For example, a longitudinal study of Chinese undergraduates reported psychological distress in 20% to 40% of students, with approximately 35% showing higher depression levels than the general population (3). Across multiple countries, approximately one-third of first-year university students screen positive for at least one common mental disorder (2). These findings underscore that the university student population bears a substantial burden of mental health problems, which can adversely affect their academic performance and long-term well-being (2, 3).

Extensive research has focused on physiological pathways, including neurobiological and hormonal mechanisms, that are believed to mediate the beneficial effects of sleep quality and physical activity on psychological well-being in young populations (4). Additional studies indicate that sleep quality and physical activity are key correlates of mood symptoms in this population (4–8). Although many investigations report positive associations, findings remain inconsistent, and even within individual studies the magnitude of the associations varies across participants. Among modifiable behaviors, sleep quality and physical activity are among the behaviors most consistently associated with anxiety and depressive symptoms and therefore constitute the primary focus of this study (4–6, 9).

In Latin America, despite a well-documented epidemiological transition that has contributed to the growing burden of noncommunicable diseases, relatively few studies have examined the joint contribution of sleep quality and physical activity to psychological well-being. Prior investigations in Chile and Brazil have demonstrated independent associations between physical activity or clusters of unhealthy behaviors and depressive symptoms (7, 8). However, these studies have not formally evaluated the potential interactive (moderating) role of physical activity in the relationship between sleep quality and internalizing symptoms. Therefore, the present study extends previous regional research by simultaneously examining sleep quality and physical activity within the same analytical framework and testing whether physical activity modifies the association between poor sleep and anxiety and depressive symptoms in a large university cohort (10, 11).

Conceptually, sleep and physical activity are tightly coupled behavioral systems: disrupted sleep may reduce next-day activity through fatigue and reduced motivation, whereas habitual activity may support sleep regulation through circadian entrainment and stress-buffering mechanisms (4). Therefore, the objective of this study was to examine the associations of sleep quality and physical activity with anxiety and depressive symptoms among university students, and to explore whether physical activity moderates the association between sleep quality and mental health symptoms. We hypothesised that (i) poor sleep quality would be associated with higher anxiety and depressive symptoms; (ii) higher physical activity would be associated with lower symptom scores in a dose–response manner; and (iii) physical activity would show a moderating (buffering) effect in the sleep–symptom relationship.

## 2 Methods

### 2.1 Study design and participants

The present study represents a cross-sectional analysis derived from the EpiHealth-UCEVA project, examining the relationships among lifestyle behaviors, physical fitness, and mental health outcomes in university students. This study adhered to the guidelines set by the Strengthening the Reporting of Observational Studies in Epidemiology (STROBE) (12). A total of 1,200 undergraduate students were invited to participate in this research. Participants were recruited from a single public university using convenience sampling through institutional emails, university websites, social media announcements, and on-campus posters and flyers. The inclusion criteria for the study were as follows: (1) aged 16–35 years, which includes the age range of those at a higher risk for incident mental health problems, and (2) required active enrollment during the 2024–2025 academic cycle; all participants completed the survey during the 2025 data-collection window. Exclusions due to incomplete data (*n* = 75, 6.3% of the invited sample) reflected missing anthropometric measurements or covariate data collected outside the online survey. The socioeconomic profile of the sample corresponded predominantly to low- to middle-socioeconomic status according to national classifications, and the majority of participants self-identified as mestizo. Data were collected from July to September 2025 (second academic semester), in Túlua, Colombia.

### 2.2 Measurements

This study used an online survey format. Before data collection, all research team members received standardized training. Participants were instructed on how to complete the survey before participation. To ensure individual responses, each participant was assigned a unique survey code. The survey could only be submitted once all items were completed. In addition, data were monitored in real time to ensure accuracy and reliability. Physical activity was assessed using the short, self-administered International Physical Activity Questionnaire (IPAQ), which captures the frequency and duration of vigorous, moderate, and walking activities performed during the previous 7 days (13). Data were processed according to the official IPAQ scoring protocol, converting each activity category into metabolic equivalent task minutes per week (MET-min/week) using standard MET coefficients (vigorous, 8.0; moderate, 4.0; walking, 3.3). Total physical activity was calculated as the sum of all domains and expressed as overall MET-min/week. Participants were classified as having low, moderate, or high physical activity according to established cutoffs (13). Adherence to the Mediterranean diet was assessed using the validated 14-item Mediterranean Diet Adherence Screener (MEDAS) (14). MEDAS was originally developed and validated in the PREDIMED trial and has been widely used in epidemiological research, demonstrating acceptable validity and reliability in European (14) and other non-Mediterranean populations (15). The questionnaire evaluates adherence to key dietary components and habits using binary (yes/no) responses, yielding a total score ranging from 0 to 14, with higher scores indicating greater adherence. Consistent with the prior literature, a score of ≥ 9 was used to define adequate adherence to the MD (14).

Anxiety and depressive symptoms were assessed using the Hospital Anxiety and Depression Scale (HADS), a widely used screening instrument for identifying anxiety and depressive symptoms in both medical and community populations (16). The scale comprises two 7-item subscales for anxiety (HADS-A) and depression (HADS-D), with each item scored from 0 to 3 and total subscale scores ranging from 0 to 21, with higher scores indicating greater severity of the symptoms. For stratified descriptive analyses, clinically significant symptoms were defined as HADS-A ≥ 11 and HADS-D ≥ 11, as supported by previous studies (17, 18). Sleep quality was assessed using the Pittsburgh Sleep Quality Index (PSQI). This validated self-report questionnaire yields a global score ranging from 0 to 21, with higher scores indicating poorer sleep quality (19). Sociodemographic characteristics were collected via self-report using a standardised questionnaire. The variables included marital status (single, married, cohabiting, and separated/other), socioeconomic status classified according to national criteria (low [levels I–II], medium [level III], or high [levels V–VI]), residence (urban or rural), race/ethnicity (Mestizo, Afro-descendant, or Indigenous/other), smoking status, and alcohol consumption, which were recorded using binary responses (yes/no). Self-reported diseases were assessed using a standardised questionnaire and categorised into respiratory, cardiovascular, metabolic, mental, and other conditions. Participants could report more than one condition each. Individuals who did not report any physician-diagnosed conditions were classified as having no disease. Standing height was measured to the nearest 0.1 cm using a calibrated stadiometer (Seca, Chino, CA, USA). Body weight was assessed using a multifrequency bioelectrical impedance analysis system (InBody 770, InBody Co. Ltd., Seoul, Korea). Body mass index (BMI) was calculated as weight in kilograms divided by height in meters squared (kg/m^2^). Waist circumference was measured in the horizontal plane midway between the lowest rib and the iliac crest.

### 2.3 Statistical analysis plan

Participant characteristics were summarised using descriptive statistics. Continuous variables are presented as mean ± standard deviation, and categorical variables are presented as frequencies and percentages. All descriptive analyses were stratified by sex and HADS-A or HADS-D scores ≥11, indicating the presence of anxiety or depression symptoms. Group comparisons were conducted separately for each sex. For continuous variables, differences between participants with and without symptoms were assessed using independent t-tests. For categorical variables, comparisons were performed using the *χ*^2^ test or Fisher’s exact test. No imputation of missing data was conducted, and the analyses were based on the available cases.

Sex-stratified analyses were conducted as exploratory analyses to assess the consistency of associations across sexes. Effect modification by sex was formally evaluated by including interaction terms in the primary regression models. Academic program and semester of enrollment were also recorded; however, these variables were not included in the final models because preliminary analyses showed no evidence of effect modification. As no statistically significant interactions were observed, the sex-stratified analyses were considered exploratory, and results from the combined models are presented as the primary findings of this study. Associations among sleep quality, physical activity, and mental health outcomes were examined using multivariable regression models.

Multivariable linear regression models were used to examine associations of sleep quality (PSQI total score) and physical activity level (low, moderate, high) with anxiety (HADS-A) and depressive symptoms (HADS-D), both treated as continuous outcomes. Physical activity was classified into low, moderate, and high levels according to the official IPAQ scoring protocol and analyzed categorically in all regression models. Hierarchical models were constructed for each outcome: Model 1 included sleep quality and physical activity; Model 2 additionally adjusted for age and sex; Model 3 further included an age × sex interaction term. Unstandardized coefficients (*β*), standardized β, 95% confidence intervals, and 2-sided *p* values were reported. Model fit was evaluated using R^2^ and adjusted R^2^, and changes in explained variance (ΔR^2^) were assessed across models. Regression assumptions were examined through residual diagnostics and multicollinearity testing. Statistical significance was defined as *p* < 0.05.

Secondary analyses examined whether physical activity moderated the association between sleep quality and internalizing symptoms. Moderation models were estimated using the PROCESS macro, version 4.0 (Model 1). Separate models were conducted for anxiety and depressive symptom scores (HADS-A and HADS-D) as continuous outcomes. Sleep quality (PSQI total score) was specified as the independent variable, physical activity level (IPAQ categories: low, moderate, high) as the moderator, and age and sex were included as covariates in all models. IPAQ categories were dummy-coded automatically within PROCESS, generating two interaction terms representing moderate and high activity levels relative to the low activity reference group. Statistical significance of moderation was evaluated using the change in explained variance (ΔR^2^) attributable to the interaction terms and the corresponding F test. Conditional effects of sleep quality on symptom scores were estimated at each level of physical activity. Bootstrapping in PROCESS was used only for confidence interval estimation and did not impute missing values. Statistical significance was defined as 2-sided *p* < 0.05. Descriptive and regression analyses were conducted in SPSS 26.0; moderation analyses were implemented using PROCESS v4.0 within SPSS.

## 3 Results

### 3.1 Characteristics according to sex and anxiety/depressive symptom groups

In this cross-sectional sample of university students, women had a higher prevalence of clinically significant anxiety symptoms than men (156/638 [24.5%] vs. 62/487 [12.7%]; *p* < 0.001), whereas depressive symptom prevalence was comparable (129/638 [20.2%] vs. 82/487 [16.8%]; *p* = 0.105), Tables 1, 2. Within both sexes, participants with anxiety or depressive symptoms exhibited significantly poorer sleep quality and higher total HADS scores compared with those without symptoms (all *p* < 0.001). Lower physical activity levels were consistently observed among symptomatic groups, particularly among women. Most sociodemographic characteristics and anthropometric measures were similar across symptom groups. Among women, depressive symptoms were additionally associated with lower adherence to the Mediterranean diet, whereas other characteristics showed no consistent differences by sex or symptom status.

**Table 1:** Participant characteristics by sex and anxiety symptoms (HADS-A ≥ 11) group.

Characteristics	Females (*n* = 638)		*p* value	Males (*n* = 487)		*p* value
	No anxiety (*n* = 482)	Anxiety (*n* = 156)		No anxiety (*n* = 425)	Anxiety (*n* = 62)	
Marital status, *n* (%)						
Single	444 (92.1)	147 (94.2)	0.480	379 (89.2)	61 (98.4)	0.003
Married	29 (6.0)	6 (3.8)		30 (7.1)	0 (0.0)	
Cohabiting	7 (1.5)	2 (1.3)		13 (3.1)	1 (1.6)	
Separated/Other	2 (0.4)	1 (0.6)		3 (0.7)	0 (0.0)	
Socioeconomic status, *n* (%)						
Low (Levels I–II)	327 (67.8)	107 (68.6)	0.286	285 (67.1)	42 (67.7)	0.450
Medium (Level III)	120 (24.9)	36 (23.1)		112 (26.4)	18 (29.0)	
High (Levels V–VI)	35 (7.3)	13 (8.3)		28 (6.6)	2 (3.2)	
Residence, *n* (%)						
Rural	65 (13.5)	17 (10.9)	0.317	70 (16.5)	12 (19.4)	0.956
Urban	417 (86.5)	139 (89.1)		355 (83.5)	50 (80.6)	
Race/ethnicity, *n* (%)						
Mestizo	426 (88.4)	135 (86.5)	0.755	360 (84.7)	53 (85.5)	0.520
Afro-descendant	5 (1.0)	3 (1.9)		13 (3.1)	2 (3.2)	
Indigenous/Other	51 (10.6)	18 (11.5)		52 (12.2)	7 (11.3)	
Self-report diseases, *n* (%)						
Respiratory	32 (6.6)	6 (3.8)	0.005	29 (6.8)	4 (6.5)	0.077
Cardiovascular	6 (1.2)	7 (4.5)		10 (2.4)	0 (0.0)	
Metabolic	41 (8.5)	19 (12.2)		26 (6.1)	4 (6.5)	
Mental	28 (5.8)	31 (19.9)		14 (3.3)	7 (11.3)	
Other conditions	17 (3.5)	7 (4.5)		8 (1.9)	4 (6.5)	
None	358 (74.3)	86 (55.1)		337 (79.5)	43 (69.4)	
Anthropometry						
Age, y	20.01 (2.93)	19.80 (3.07)	0.390	20.82 (4.08)	20.30 (3.29)	0.340
Body mass index, kg/m^2^	23.88 (4.09)	24.53 (4.94)	0.120	24.05 (4.40)	23.16 (4.22)	0.150
Waist circumference, cm	72.16 (8.30)	73.75 (9.98)	0.060	78.48 (10.07)	77.22 (8.71)	0.360
Sleep quality						
PSQI total score	5.60 (2.80)	8.20 (3.21)	<0.001	5.02 (2.88)	8.03 (3.58)	<0.001
Diet						
MEDAS score	5.52 (1.94)	5.79 (2.01)	0.14	5.98 (1.84)	5.89 (2.44)	0.74
≥9 High adherence, *n* (%)	40 (8.3)	15 (9.6)	0.588	35 (8.2)	9 (14.5)	0.794
Alcohol intake, *n* (%)	8 (1.7)	1 (0.6)	0.775	11 (2.6)	6 (9.7)	0.003
Mental health						
HADS total score	14.14 (3.95)	21.37 (3.96)	<0.001	13.26 (3.75)	21.00 (3.68)	<0.001
Physical activity levels, *n* (%)						
Low	235 (49.0)	92 (59.4)	0.110	99 (23.6)	27 (43.5)	0.002
Moderate	107 (22.3)	28 (18.1)		116 (27.6)	11 (17.7)	
High	138 (28.7)	35 (22.6)		205 (48.8)	24 (38.7)	
MET-min/wk. score	1387.59 (1522.18)	1095.54 (1415.51)	0.030	2266.11 (1692.19)	1641.73 (1627.67)	0.020

Values are presented as mean (SD) for continuous variables and number (percentage) for categorical variables. Anxiety symptoms were defined as HADS-A ≥ 11. *p* values correspond to overall comparisons across categories within each characteristic (*χ*^2^/Fisher), within each sex. Fisher exact tests were used when expected cell counts were <5. MET-min/wk, metabolic equivalent–minutes per week; PSQI, Pittsburgh Sleep Quality Index; HADS, Hospital Anxiety and Depression Scale; MEDAS, 14-item Mediterranean Diet Adherence Screener. Percentages are column percentages within sex and symptom group.

**Table 2:** Participant characteristics by sex and depressive symptoms (HADS-D ≥ 11) group.

Characteristics	Females (*n* = 638)		*p* value	Males (*n* = 487)		*p* value
	No depression (*n* = 509)	Depression (*n* = 129)		No depression (*n* = 405)	Depression (*n* = 82)	
Marital status, *n* (%)						
Single	471 (92.5)	120 (93.0)	0.239	361 (89.1)	79 (96.3)	0.931
Married	29 (5.7)	6 (4.7)		28 (6.9)	2 (2.4)	
Cohabiting	6 (1.2)	3 (2.3)		14 (3.5)	0 (0.0)	
Separated/Other	3 (0.6)	0 (0.0)		2 (0.5)	1 (1.2)	
Socioeconomic status, *n* (%)						
Low (Levels I–II)	351 (69.0)	83 (64.3)	0.098	271 (66.9)	56 (68.3)	0.520
Medium (Level III)	119 (23.4)	37 (28.7)		106 (26.2)	24 (29.3)	
High (Levels V–VI)	39 (7.7)	9 (7.0)		28 (6.9)	2 (2.4)	
Residence, *n* (%)						
Rural	65 (12.8)	17 (13.2)	0.776	69 (17.0)	13 (15.9)	0.820
Urban	444 (87.2)	112 (86.8)		336 (83.0)	69 (84.1)	
Race/ethnicity, *n* (%)						
Mestizo	446 (87.6)	115 (89.1)	0.421	347 (85.7)	66 (80.5)	0.373
Afro-descendant	7 (1.4)	1 (0.8)		13 (3.2)	2 (2.4)	
Indigenous/Other	56 (11.0)	13 (10.1)		45 (11.1)	14 (17.1)	
Self-report diseases, *n* (%)						
Respiratory	35 (6.9)	3 (2.3)	0.300	26 (6.4)	7 (8.6)	0.102
Cardiovascular	9 (1.8)	4 (3.1)		6 (1.5)	4 (4.9)	
Metabolic	47 (9.2)	13 (10.1)		24 (5.9)	6 (7.4)	
Mental	45 (8.8)	14 (10.9)		14 (3.5)	7 (8.6)	
Other conditions	17 (3.3)	7 (5.4)		9 (2.2)	3 (3.7)	
None	356 (69.9)	88 (68.2)		326 (80.5)	54 (66.7)	
Anthropometry/body composition						
Age, y	19.96 (2.83)	19.96 (3.44)	0.99	20.89 (4.08)	20.08 (3.48)	0.070
Body mass index, kg/m^2^	23.94 (4.17)	24.41 (4.85)	0.28	23.88 (4.33)	24.21 (4.66)	0.560
Waist circumference, cm	72.30 (8.53)	73.52 (9.58)	0.13	78.19 (9.94)	78.93 (9.78)	0.560
Sleep quality						
PSQI total score	6.04 (2.93)	7.01 (3.67)	<0.001	5.30 (3.12)	5.93 (3.40)	<0.001
Diet						
MEDAS score	5.65 (2.01)	5.34 (1.76)	0.10	5.99 (1.93)	5.84 (1.90)	0.510
≥9 High adherence, *n* (%)	52 (10.2)	3 (2.3)	0.006	38 (9.4)	6 (7.3)	0.256
Alcohol intake, *n* (%)	7 (1.4)	2 (1.6)	0.122	13 (3.2)	4 (4.9)	0.866
Mental health						
HADS total score	14.65 (4.38)	20.88 (4.28)	<0.001	13.32 (4.15)	18.82 (3.56)	<0.001
Physical activity levels, *n* (%)						
Low	253 (49.9)	74 (57.8)	0.023	95 (23.8)	31 (37.8)	0.001
Moderate	112 (22.1)	23 (18.0)		108 (27.0)	19 (23.2)	
High	142 (28.0)	31 (24.2)		197 (49.3)	32 (39.0)	
MET-min/wk. score	1347.92 (1503.31)	1191.05 (1491.11)	0.290	2274.09 (1699.79)	1755.11 (1615.20)	0.020

Values are presented as mean (SD) or number (percentage). Depressive symptoms were defined as HADS-D ≥ 11. *p* values correspond to overall comparisons across categories within each characteristic (*χ*^2^/Fisher), within each sex. Fisher exact tests were used when expected cell counts were <5. MET-min/wk, metabolic equivalent–minutes per week; PSQI, Pittsburgh Sleep Quality Index; HADS, Hospital Anxiety and Depression Scale; MEDAS, 14-item Mediterranean Diet Adherence Screener. Percentages are column percentages within sex and symptom group.

### 3.2 Associations of sleep quality and physical activity with mood symptoms

In adjusted analyses (Table 3), poorer sleep quality was strongly associated with higher anxiety symptom scores (*B* = 0.59; 95% CI, 0.53–0.66; standardized *β* = 0.48; *p* < 0.001) and modestly associated with higher depressive symptoms (*B* = 0.10; 95% CI, 0.05–0.15; standardized *β* = 0.11; *p* < 0.001). For anxiety, moderate physical activity was independently associated with lower symptom levels compared with low activity (*B* = −0.57; 95% CI, −1.10 to −0.05; *p* = 0.033), whereas high physical activity was not statistically significant after adjustment. In contrast, for depressive symptoms, high physical activity was significantly associated with lower scores (*B* = −0.62; 95% CI, −1.02 to −0.21; *p* = 0.003), while moderate activity was not. Age was inversely associated with both anxiety and depressive symptoms. Sex was associated with anxiety but not depression. Full regression results for all models, including crude and interaction analyses, are presented in Supplementary eTables 1, 2.

**Table 3:** Associations of sleep quality and physical activity with anxiety and depressive symptoms.

Predictor	Anxiety symptoms, (HADS-A)			Depressive symptoms, (HADS-D)		
	B (95% CI)	Std β	*p*	B (95% CI)	Std β	*p*
Sleep quality						
PSQI total (points)	0.59 (0.53 to 0.66)	0.48	<0.001	0.10 (0.05 to 0.15)	0.11	<0.001
IPAQ category						
Low	Ref			Ref		
Moderate	−0.57 (−1.10 to −0.05)	−0.15	0.033	−0.41 (−0.86 to 0.03)	−0.14	0.069
High	−0.37 (−0.84 to 0.11)	−0.09	0.131	−0.62 (−1.02 to −0.21)	−0.21	0.003
Age (years)	−0.08 (−0.14 to −0.02)	−0.07	0.006	−0.07 (−0.12 to −0.02)	−0.08	0.008
Sex, Male (vs Female)	0.77 (0.35 to 1.19)	0.20	<0.001	−0.11 (−0.46 to 0.25)	−0.04	0.557

Values are unstandardized regression coefficients (B) with 95% confidence intervals (CIs). Standardized β (Std). Model was adjusted for age and sex. Physical activity categories were derived from the IPAQ scoring protocol (low, moderate, high). Anxiety and depressive symptoms were assessed using the Hospital Anxiety (HADS-A) and Depression Scale (HADS-D). Sleep Quality indicates total score on the Pittsburgh Sleep Quality Index, with higher scores reflecting poorer sleep quality. Two-sided *p* values are reported. Model fit: Anxiety: *R*^2^ = 0.27; Depression: *R*^2^ = 0.03.

### 3.3 Moderation analysis

Moderation analyses were conducted to examine whether physical activity modified the association between sleep quality and internalizing symptoms, adjusting for age and sex. For anxiety symptoms (HADS-A), the overall model explained R^2^ = 0.27 of the variance (F₇,₁₁₀₉ = 58.26; *p* < 0.001). Poorer sleep quality was significantly associated with higher anxiety symptoms (*B* = 0.68; 95% CI, 0.58–0.78; *p* < 0.001). The overall interaction was not statistically significant (ΔR^²^ = 0.0033; *p* = 0.08). However, the interaction term for high (vs low) physical activity reached statistical significance (*B* = −0.15; 95% CI, −0.30 to −0.01; *p* = 0.04), suggesting a possible attenuation of the sleep–anxiety association at higher activity levels. Bootstrap confidence interval estimates (5,000 samples) were consistent with the main results.

For depressive symptoms, the overall model explained R^2^ = 0.03 (F₇,₁₁₀₉ = 5.18; *p* < 0.001), indicating that only a small proportion of variance in depressive symptoms was explained. Poorer sleep quality was associated with higher depressive symptoms (*B* = 0.10; 95% CI, 0.05–0.15; *p* < 0.001). The overall interaction was not statistically significant (ΔR^²^ = 0.0029; *p* = 0.19), and neither interaction term reached statistical significance (all *p* > 0.05). The moderating effects of physical activity on the associations between sleep quality and anxiety symptoms and between sleep quality and depressive symptoms are presented in Supplementary Figures S1, S2.

## 4 Discussion

Among university students, physical activity may serve as a protective modifier of sleep-related anxiety vulnerability, but its buffering role appears more limited for depressive symptoms. These findings are consistent with prior studies in student populations showing associations between sleep disturbances and adverse mood symptoms (9–11, 20–22). Multiple studies have indicated that anxiety and depressive symptoms are highly prevalent among university students globally. Nevertheless, prevalence estimates vary substantially across geographic contexts, assessment tools, and methodological approaches. In the present study, clinically significant symptoms were observed in 218/1125 (19.4%) for anxiety and 211/1125 (18.8%) for depression. Consistent with these findings, systematic reviews suggest that approximately one-third of university students experience elevated symptoms of depression or anxiety (23). For example, one meta-analysis estimated a global pooled prevalence of depressive disorders among university students of approximately 30.6%, substantially higher than that reported for the general population (22). Similarly, an umbrella review synthesising evidence from 25 systematic reviews reported a median prevalence of anxiety of approximately 32%, with estimates ranging from 7% to 55% (24). For instance, a HADS-based survey in Iraq (Baghdad) reported that 45.9% of medical/pharmacy students had depression symptoms (plus 24.8% “borderline” cases) and 52.1% had anxiety symptoms (with an additional 20.1% borderline) (25). Differences may reflect the screening instrument, cutoffs, academic calendar timing, and regional/contextual factors.

Similarly, evidence from Latin America, much of it derived from studies using the HADS or comparable instruments, consistently indicates a high prevalence of anxiety and depression symptoms among university students. A 2021 systematic review and meta-analysis from Brazil, including 44 studies and 37,486 undergraduates, reported pooled prevalence estimates of 37.7% for anxiety and 28.5% for depression (23). Extensive observational studies have confirmed these findings. For instance, a 2024 cross-sectional survey conducted in southern Brazil (the Respire study; *n* = 5,112) using the HADS found that 34.3% of students reported moderate-to-severe anxiety symptoms and 24.3% reported moderate-to-severe depressive symptoms (26).

Physical activity showed a modest moderating pattern for anxiety, with attenuation of the sleep–anxiety association at higher activity levels, whereas no moderation was observed for depressive symptoms. Physical activity has consistently been reported to exert antidepressant effects (27). In general, physical activity has been repeatedly linked to better psychological well-being among young adults (27, 28), and our moderation analyses suggest that lower activity levels may be associated with differences in the magnitude of the association between poor sleep quality and mental health symptoms. Notably, the magnitude of the interaction effects was modest, indicating that sleep quality and physical activity likely represent complementary rather than substitutive correlates of poor mental health. Some hypothesised mechanisms may explain the association between physical activity, better sleep quality, anxiety, and depression. Physiologically, both measures are associated with a lower risk of neuroinflammation and increased production and secretion of neurotrophins, such as the brain-derived neurotrophic factor (29, 30). A recent meta-analysis encompassing 49 prospective cohort studies and 1,837,794 person-years revealed that individuals engaging in high levels of physical activity had a 17% reduced likelihood of experiencing depression (OR = 0.83, CI = 0.79, 0.88) compared with those with low physical activity (27). Other meta-analyses have similarly indicated that low physical activity is associated with an increased risk of depression (29). Furthermore, from a psychosocial standpoint, engaging in physical exercise can mitigate symptoms of depression, anxiety, and stress while enhancing overall well-being and sleep quality (31, 32). Although prior evidence suggests potential roles of diet, body composition and physical fitness in mental health, these dimensions were not central to the present analyses and require further targeted investigation (33, 34).

Several limitations of this study should be considered when interpreting the results. First, the cross-sectional nature of the study prevents conclusions about causality or the sequence of events. Consequently, it is not possible to determine whether poor sleep quality and low physical activity contribute to anxiety and depression symptoms or whether the presence of these symptoms leads to adverse lifestyle changes. This limitation is particularly important in light of our moderation analyses, which suggested a modest attenuation of the sleep–anxiety association at higher levels of physical activity but no evidence of moderation for depressive symptoms, and therefore do not support causal inference. However, from a public health perspective, while causality cannot be inferred, the observed associations support consideration of integrated screening strategies addressing sleep quality and physical activity within university health promotion programs, subject to validation in longitudinal and intervention studies. Second, the use of a convenience sample derived from a pilot study limits the generalisability of the results. Third, although validated instruments were used, the assessment of anxiety and depressive symptoms relied on self-report scales intended for symptom screening rather than clinical diagnoses. In addition, symptoms were reported for the 2 weeks preceding data collection and may have been influenced by transient academic or psychosocial stressors, potentially introducing temporal bias. The current findings should also be interpreted within the broader post–COVID-19 context, during which substantial disruptions in sleep patterns, physical activity behaviors, and mental health were observed globally (35, 36). Residual behavioral and psychosocial effects from this period may partly contribute to the elevated symptom prevalence detected in the present cohort. Fourth, we acknowledge that the IPAQ, particularly when self-administered, may overestimate physical activity levels. To mitigate this limitation, we applied the official IPAQ scoring protocol and categorical classification (low, moderate, high) rather than relying solely on continuous MET-min/week estimates. Because sleep quality, physical activity, and symptoms were measured contemporaneously, the moderation analyses should be interpreted as an association decomposition rather than evidence of a causal pathway. Reverse causation (e.g., symptoms influencing sleep and activity) and unmeasured confounding may produce interaction effects in cross-sectional data.

Although this study had some limitations, it had significant strengths. To our knowledge, this is one of the pioneering studies to explore the combined effects of sleep quality and physical activity on anxiety and depressive symptoms among university students in Latin America. By employing both regression and moderation methods, the analyses provided deeper insights into how lifestyle factors might be interconnected in their impact on mental health. These results highlight the interconnectedness of sleep, physical activity, and mental health among university students, stressing that causal conclusions cannot be drawn. Overall, moderation results should be viewed as a statistical breakdown of associations rather than proof of the causal pathways. Future longitudinal and intervention studies are necessary to elucidate these temporal relationships and determine whether enhancing sleep quality and physical activity together leads to improved mental health outcomes in various university settings.

## 5 Conclusion

In this cross-sectional analysis, poorer sleep quality was robustly associated with higher anxiety and depressive symptom levels. Physical activity demonstrated a modest moderating pattern for anxiety but not for depressive symptoms. These findings reinforce the central role of sleep quality in student mental health and suggest that physical activity may buffer anxiety-related risk under conditions of poor sleep. Prospective and interventional studies are warranted to clarify directionality and causal mechanisms.

## Data Availability

The raw data supporting the conclusions of this article will be made available by the authors, without undue reservation.
