# Supplementary material for: Associations of sleep quality and physical activity with anxiety and depressive symptoms among university students: a cross-sectional study
Source: Front Public Health. 2026 Mar 19;14:1799311. doi: 10.3389/fpubh.2026.1799311 (PMC13044136; doi:10.3389/fpubh.2026.1799311)
Supplement: Supplementary file 1 [file Data_Sheet_1.pdf]

Supplementary Material

eTable 1. Multivariable Linear Regression Models for Anxiety and Symptoms

Predictor	Model 1 β	Model 2 β	Model 3 β
PSQI total (points)	0.60***	0.59***	0.60***
Moderate PA	−0.75**	−0.57*	−0.58*
High PA	−0.61*	−0.37	−0.36
Age	—	−0.08*	−0.06
Sex	—	0.77***	1.81
Age × Sex	—	—	−0.05

Hierarchical models were constructed for each outcome: Model 1 included sleep quality and physical activity; Model 2 additionally adjusted for age and sex; Model 3 further included an age × sex interaction term. R^2: M1 = 0.250; M2 = 0.266; M3 = 0.266; ΔR^2 (M1→M2) = 0.015 ($P < .001$); ΔR^2 (M2→M3) = ns

eTable 2. Multivariable Linear Regression Models for Depressive Symptoms

Predictor	Model 1 β	Model 2 β	Model 3 β
PSQI total (points)	0.094***	0.10***	0.10***
Moderate PA	−0.42	−0.41	−0.41
High PA	−0.61**	−0.62**	−0.62**
Age	—	−0.07*	−0.08*
Sex	—	−0.11	−0.59
Age × Sex	—	—	0.02

Hierarchical models were constructed for each outcome: Model 1 included sleep quality and physical activity; Model 2 additionally adjusted for age and sex; Model 3 further included an age × sex interaction term. R^2: M1 = 0.022; M2 = 0.029; M3 = 0.029; ΔR^2 (M1→M2) = 0.006 ($P = .027$); ΔR^2 (M2→M3) = ns

Figure S1. Association Between Sleep Quality and Anxiety Symptoms According to Physical Activity Level.

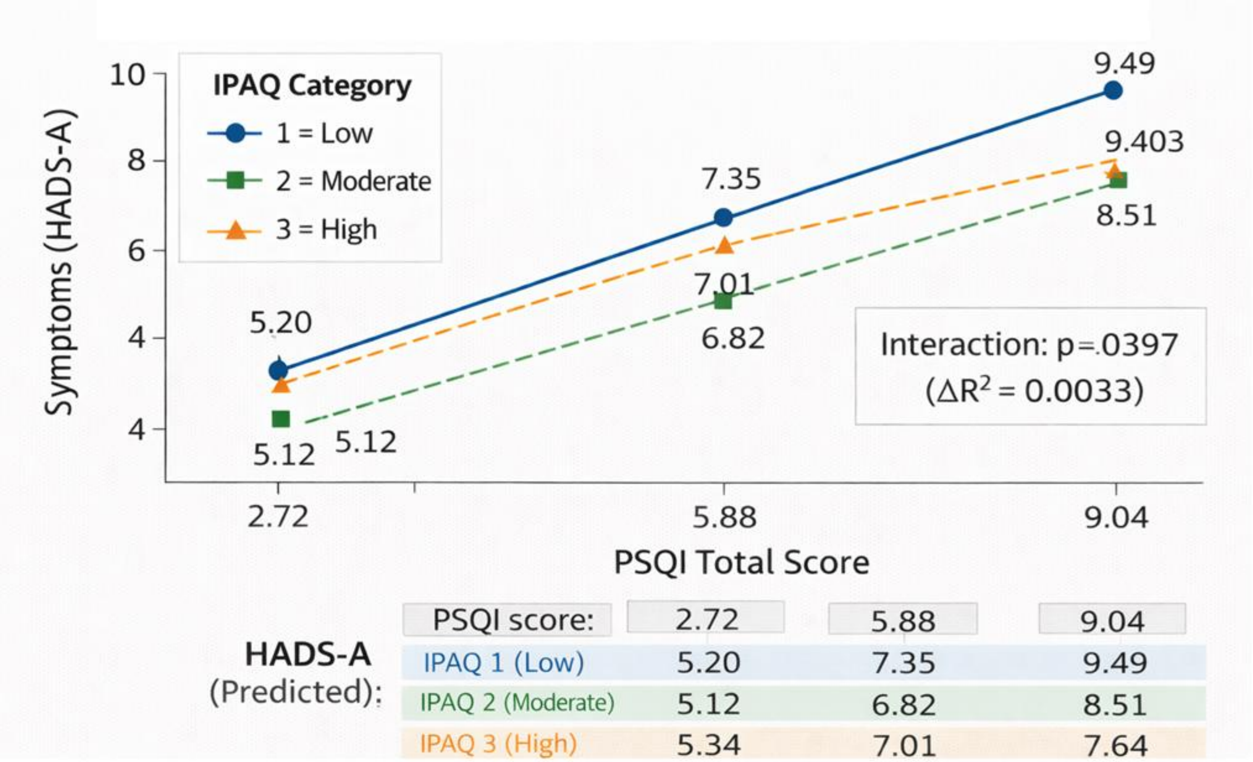

Lines represent adjusted linear regression estimates stratified by physical activity category. Higher physical activity levels attenuated the strength of the association.

Figure S2. Association Between Sleep Quality and Depressive Symptoms According to Physical Activity Level

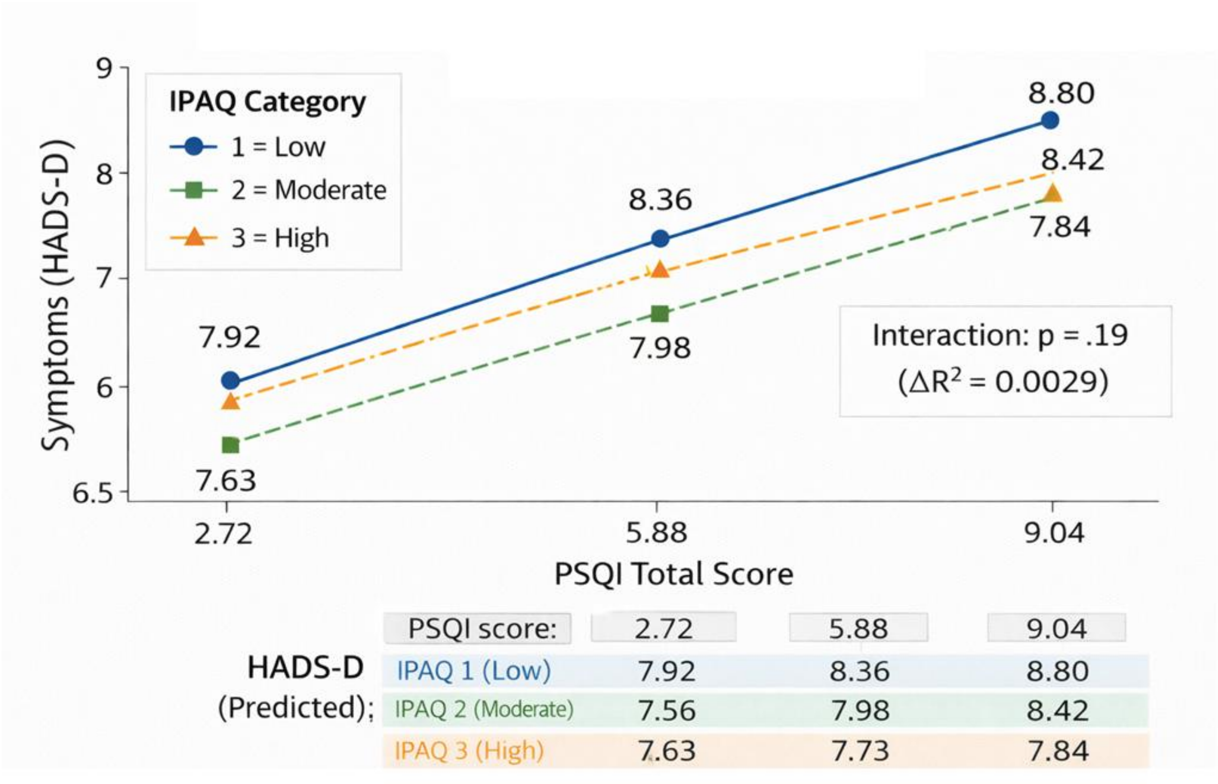

Physical activity did not significantly modify the association between sleep quality and depressive symptoms.
